# Bayesian Updating of Fatigue Crack Growth Parameters for Failure Prognosis of Miter Gates

**DOI:** 10.3390/ma18051172

**Published:** 2025-03-06

**Authors:** Anita Brown, Brian Eick, Travis Fillmore, Hai Nguyen

**Affiliations:** 1 Coastal and Hydraulics Laboratory (CHL), Engineer Research and Development Center (ERDC), United States Army Corps of Engineers (USACE), Vicksburg, MS 39180, USA; travis.b.fillmore@erdc.dren.mil; 2 Construction Engineering Research Laboratory (CERL), Engineer Research and Development Center (ERDC), United States Army Corps of Engineers (USACE), Champaign, IL 61826, USA; brian.a.eick@erdc.dren.mil (B.E.); hai.d.nguyen@erdc.dren.mil (H.N.)

**Keywords:** miter gates, fatigue cracking, Paris’ law, numerical modeling, digital image correlation (DIC), stress intensity factor (SIF)

## Abstract

Navigable waterways play a vital role in the efficient transportation of millions of tons of cargo annually. Inland traffic must pass through a lock, which consists of miter gates. Failures and closures of these gates can significantly disrupt waterborne commerce. Miter gates often experience fatigue cracking due to their loading and welded connections. Repairing every crack can lead to excessive miter gate downtime and serious economic impacts. However, if the rate of crack growth is shown to be sufficiently slow, e.g., using Paris’ law, immediate repairs may be deemed unnecessary, and this downtime can be avoided. Paris’ law is often obtained from laboratory testing with detailed crack measurements of specimens with relatively simple geometry. However, Paris’ law parameters for an in situ structure will likely deviate from those predicted from physical testing due to variations in loading and materials and a far more complicated geometry. To improve Paris’ law parameter prediction, this research proposes a framework that utilizes (1) convenient vision-based tracking of crack evolution both in the laboratory and the field and (2) numerical model estimation of stress intensity factors (SIFs). This study’s methodology provides an efficient tool for Paris’ law parameter prediction that can be updated as more data become available through vision-based monitoring and provide actionable information about the criticality of existing cracks.

## 1. Introduction

The United States has an extensive network of lock and dam sites, many of which are managed by the US Army Corps of Engineers (USACE). At such sites, critical structures like miter gates play a pivotal role in facilitating commercial activities and logistics. They are vital for maintaining navigable waterways for cargo transportation, thus enhancing both global and local economies. However, these miter gates face challenges due to cyclic loading from operational demands and environmental factors, with fatigue cracks being a common damage mode. This damage may inhibit miter gate operation, which will cause the lock to shut down, thus blocking river traffic and harming the economy. While inspections may identify fatigue damage before criticality, the lack of visibility of submerged critical components and the difficult conditions for underwater evaluations require fully draining a lock chamber to facilitate a single inspection, making inspections infrequent. As a result of these complications, any cracks identified during inspections are often repaired immediately. However, in the case that the crack’s future growth is not critical to structural performance, this practice can lead to unnecessary and costly outages, which may cost as much as USD 3 million per day [1].

An alternative to immediate repair is fatigue crack growth assessment. Due to the nature of the inspection data providing crack lengths over periods of time, Paris’ law was selected to predict the fatigue crack growth rates. Paris’ law describes a log-linear relationship between the stress intensity factor (SIF) and the rate of crack growth. The resulting Paris’ law parameters incorporate the effects of microstructure, environmental conditions, mechanical loading, and material properties. Parameterizing this law typically involves obtaining data from controlled fatigue experiments, such as those performed by Roux-Langlois et al. [2], Wang et al. [3], Yang et al. [4], and Wu and Ni [5]. Sobczyk [6] used Paris’ law as a base to stochastically model fatigue crack growth under random loading based on experimental results. Mikulski and Lassen [7] used Paris’ law to determine the growth parameters during the crack propagation phase in fillet-welded steel joints with cracks initiating from the weld toe. In fatigue testing, measurement methods for crack length include the use of cameras and zoom microscopes to define the displacement fields around the crack tip. Although the SIF can be calculated from the displacement field, a threshold value below the fracture toughness is sometimes utilized. Roux-Langlois et al. [2] provide a detailed review of methods used to estimate the position of the crack tip and determine SIFs. However, many of these methods are resource-intensive, significantly increasing the cost of performing fatigue testing, or require assumptions to be made.

Numerical modeling allows for crack simulation and SIF calculation using techniques such as XFEM/GFEM [8,9]. Further, Bayesian-inference-based methods allow for improving the estimation of crack size over time. Researchers have inferred crack size using digital image correlation (DIC) ([10,11,12,13]), strain gages [14], accelerometers [15], and acoustic emission measurements ([16,17]). Further, DIC has been used for the prediction of SIF parameters and the development of crack propagation laws [18,19]. Yet, estimating SIFs using DIC requires high-density speckle application and appropriately sensitive cameras, which may be difficult for large test specimens. In the context of an in situ miter gate, Paris’ law parameters have been updated using Bayesian methods in a synthetic example problem using strain gage observations [14], and stochastic crack growth has been modeled for reliability [20]. However, this research considers synthetic example problems, assumes constant crack propagation values for experimental data, and can require difficult-to-install contact sensors.

This research proposes a method for estimating Paris’ law parameters for an in situ structure by leveraging the estimates obtained from experimental testing along with easily obtained data of crack growth rates from the in situ structure. The proposed methodology is agnostic to the method in which the crack growth data are acquired; however, this research proposes using imagery from inexpensive cameras along with numerical modeling to estimate crack growth rates and SIFs. To that end, in this study, DIC is leveraged on images of cracks propagating on the laboratory specimens. For the in situ structure, images are collected from a remotely operated vehicle (ROV) during period inspections, and the growth of a known crack is measured based on known distances between features. Further, due to the complicated geometry of both the laboratory specimens and the in situ structure, the proposed method leverages numerical modeling and the extended finite element method (XFEM) to determine the SIFs at each measured crack length, which is a necessary parameter for Paris’ law. In an example problem, the proposed methodology is applied to the estimation of Paris’ law parameters for twelve full-scale laboratory specimens under constant-amplitude cyclic loading from the tests performed by Eick et al. [21], which replicate common weld details found in miter gates. Then, Bayesian methods are used to update the Paris’ law parameters for an in situ crack monitored over several months for the miter gate at The Dalles, Oregon, USA. Through this comprehensive methodology, our research seeks to significantly enhance the predictability, management, and maintenance frameworks of miter gates by tuning the crack growth parameters based on real-world data, thereby ensuring the continued safety and efficiency of lock and dam systems essential for navigable waterways. This endeavor is especially aimed at addressing the multifaceted challenges presented by structures under cyclic loading with complex weld geometries and varying environmental conditions, thereby enhancing the structural integrity and resilience of crucial infrastructure.

## 2. Materials and Methods

### 2.1. Methodology

**Figure 1:**
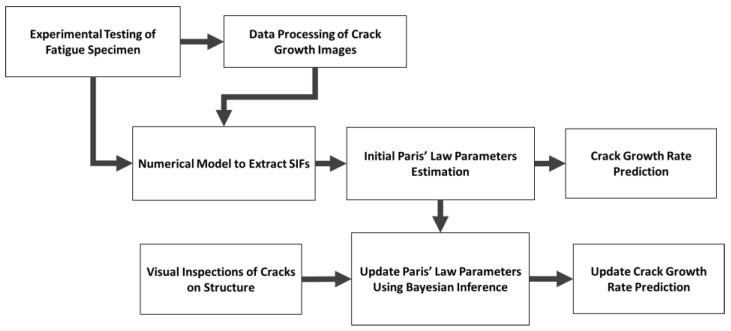
Methodology for determining crack growth parameters.

This study proposes a method for improving the prognosis of future crack growth. It employs the images captured during the full-scale testing of the twelve cruciform specimens (Eick et al. [21]), in conjunction with digital image correlation and numerical modeling, to probabilistically estimate the Paris’ law parameters for this specific loading configuration. These estimates are then refined through Bayesian updating, incorporating data of crack growth rates collected from an in situ structure and exploring an additional loading scenario. This methodology is illustrated as a flowchart in Figure 1. The methodology developed in this paper is generalizable to any structure; however, the specific results presented herein are expected to only be applicable to miter gates constructed with ASTM A572 Grade 50 steel and the equivalent ASTM A709 Grade 50 steel, which is expected to consist of most miter gates constructed in the U.S. in the last 30 years.

#### 2.1.1. Determination of Paris’ Law Parameters

Paris’ law is a commonly used approach to estimate the rate of steady crack growth for a loading cycle as a function of the change in the state of stress at the crack tip in ([22,23]). The law is stated as

(1) $$\frac{da}{dN}=C{\left(\Delta K\right)}^{m},$$

where *a* is the crack length and *N* is the number of load cycles, *C* and *m* are material parameters, and Δ*K* is the change in the SIF for a load cycle.

Paris’ law requires an accurate estimate of the SIFs at the crack tip, which can be challenging to calculate for a real structure with complicated geometry and loading. SIFs are dependent on the mode of cracking being experienced, where mode I is related to an opening crack, and modes II and III are for in- and out-of-plane shearing, respectively. With the cracks common to miter gates, some combination of these cracking modes is typical. For these mixed-mode cracks, it is common to use a combination of the respective SIFs to generate an equivalent SIF, *K_eq_*. These approaches can vary in complexity. Sajith et al. [24] discuss a variety of approaches used to calculate *K_eq_* for mixed-mode cracking. The most commonly used approach for Mode I and II dominant cracks is the energy approach, which is calculated as a magnitude using the Pythagorean theorem ([25]).

(2) $$K_{eq}={\left(K_{I}^{2}+K_{II}^{2}\right)}^{\frac{1}{2}},$$

where *K_I_* and *K_II_* are the SIFs for mode I and mode II cracks, respectively. The use of this definition relies on the assumption that the SIF associated with Mode III cracking is negligible.

When Mode III does contribute to mixed-mode cracking, the equivalent SIF can be expressed to account for the additional torsional behavior ([26,27,28]). This is expressed as

(3) $$K_{eq}=\frac{K_{I,II}\left(1+2\nu \right)+\sqrt{K_{I,II}^{2}{\left(1-2\nu \right)}^{2}+4K_{III}^{2}}\;}{2},$$

where *ν* is Poisson’s ratio and *K_I,II_* is

(4) $$K_{I,II}=\frac{0.83K_{I}+\sqrt{0.4489K_{I}^{2}+3K_{II}^{2}}\;}{1.5},$$

Although often treated as constants, it is commonly understood that the material parameters associated with Paris’ law, *C* and *m*, and the rate of crack growth are dependent on the loading conditions and geometry of the specimen which are linked to the load ratio, where a load ratio of −1 indicates a reversal of load, 0 indicates zero-compression fatigue, and 1 indicates a constant load ([23,29,30]). With knowledge of changes in the SIFs over a loading cycle, the properties of logarithms can be used to linearize (1) as follows:

(5) $$\log\left(\frac{da}{dN}\right)=\log C+m\log\Delta K,$$

where logC acts as the *y*-intercept and *m* acts as the slope. Estimation of the parameters log *C* and *m* is typically completed using standard linear regression on crack growth data. Under the assumptions of standard linear regression, each parameter can be described as a normally distributed random variable with a mean and standard deviation based on the variability of the collected data.

#### 2.1.2. Updating of Paris’ Law Parameters

Bayesian inference was employed to further update these parameters using the additional data obtained from the in-service structure. Bayesian inference relies on computing the posterior probability from Bayes theorem, written as

(6) $$\mathrm{Pr}\left(\theta |Y\right)=\frac{\mathrm{Pr}\left(Y|\theta \right)\mathrm{Pr}\left(\theta \right)}{\mathrm{Pr}\left(Y\right)},$$

where $\mathrm{Pr}\left(\theta \right)$ is the prior probability for the parameters θ, $\mathrm{Pr}\left(Y|\theta \right)$ is the likelihood of the observations *Y* given θ, $\mathrm{Pr}\left(Y\right)$ is the marginal probability of the observations, and $\mathrm{Pr}\left(\theta |Y\right)$ is the posterior probability of θ given the observed dataset ([31,32]). The normal distributions describing the Paris’ law parameters obtained from the simple linear regression acted as the prior probability. The new data obtained from the crack growth in the real miter gate were used to update this information through the marginal probability and a likelihood function, which checked the compatibility between the new observations and the initial parameter estimates. The resulting posterior probability updated the description of the Paris’ law parameters given the introduction of the observed crack growth. In this study, the marginal posterior distribution of each parameter was approximated using Markov chain Monte Carlo (MCMC) simulations. A No-U-Turn sampler (NUTS), a type of Hamiltonian Monte Carlo algorithm, was applied to generate the posterior samples that defined the updated parameters. This was completed using the PyMC, a Python probabilistic programming library [33].

#### 2.1.3. Finding $\frac{da}{dN}$ Through Laboratory Testing and Digital Image Correlation (DIC)

Paris’ law parameters are often obtained through laboratory testing of representative specimens. For the proposed methodology, crack propagation data can be obtained by any appropriate means. For this study, DIC is used on images taken during laboratory testing to extract the necessary information. DIC was selected for image processing because, from the experimental setup, it was unclear whether the crack initiation and position of the crack tip would be visibly obvious. To enable DIC, the laboratory specimens had a speckle pattern applied, and images were regularly taken of the test specimen at maximum and minimum loading.

**Figure 2:**
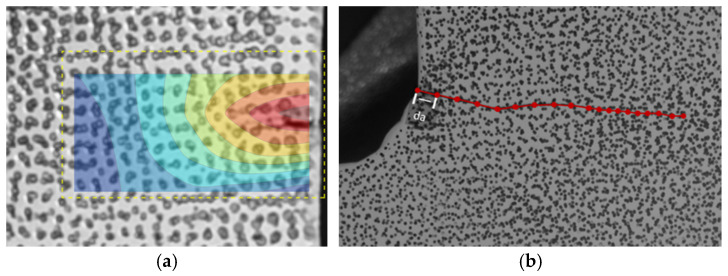
Tracking crack progression by (**a**) determining crack initiation and location of the crack tip using strain field generated using DIC and (**b**) measuring the length of the crack over cycles.

The images collected during testing were processed to identify the cycle number when the crack initiated and to track the crack length and number of cycles elapsed during subsequent images. Crack initiation and the location of the crack tip were identified using DIC by overlaying the calculated strain field on the test specimen images. The images were rectified using homography, the process of projective transformation that maps colinear points between two planes using a mapping matrix that allows for forward and backward transformation between image spaces [34], so that crack length could be directly measured per loading cycle. Instances of crack growth were tracked by discretizing the crack as line segments using labeled data points. Crack lengths were only able to be extracted once the crack reached a visible length as no microscopic equipment was used. The tracking of the crack tip and determination of crack length are illustrated in Figure 2. Consequently, a set of loading cycles, discretized crack length segments along the crack path, and crack length orientations are obtained. These allowed the direct calculation of $\frac{da}{dN}$ for Paris’ law.

#### 2.1.4. Finding $\frac{da}{dN}$ Through Analysis of Inspection Images

**Figure 3:**
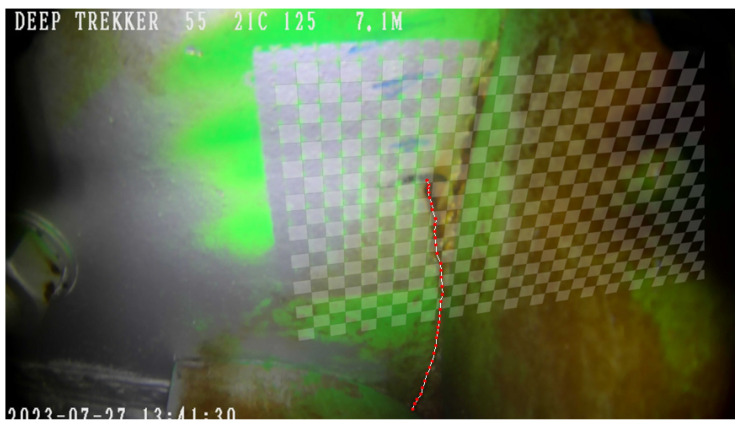
Selecting pixel coordinates to discretize the crack and determine total crack length.

The images of cracks collected from the ROV inspections were compared to identify changes in crack length between periods of inspection. In order to standardize the perspective of the images and directly compare crack lengths, homography was used to project the images into the same plane using common points identified between images. Crack lengths were tracked by discretizing the crack and converting the pixel coordinates of each point along the crack into measurements of length as shown in Figure 3. A gridded pattern of known distances that was painted in the area allowed for this conversion. Cycle counts were estimated based on the frequency of operations that occur between inspections. This allowed for an estimation of $\frac{da}{dN}$ for updating Paris’ law.

#### 2.1.5. Finding ∆*K* Through Numerical Modeling

For large-scale structures, or even large lab specimens with complicated geometry, determining ΔK for a load cycle is challenging. While DIC was leveraged in this study, the size of the specimens and uncertainty on where cracks would occur were such that the images did not have high enough resolution to calculate SIFs. Rather, a numerical model of the structure or test specimen’s geometry, with the crack geometry extracted using DIC, was used to determine the SIFs at the crack tip for each increment in growth. Employing Finite Element Modeling (FEM) and the Extended Finite Element Method (XFEM) for the computational modeling of fatigue crack growth, particularly in computing the SIF, allows for highly accurate simulations that are critical for assessing the structural integrity and fatigue life of engineering components. XFEM enables the simulation of crack propagation without the need for mesh compatibility traditionally required in standard finite element methods, thereby preserving computational resources while maintaining high precision. This feature is particularly beneficial in handling complex crack geometries or in cases where crack paths are unpredictable ([35,36,37,38,39,40,41]).

The crack geometry was modeled in Abaqus 2021 using the built-in XFEM capabilities, which model the crack as shell geometry, using the coordinates extracted from the DIC of the experimental testing images. This process requires the assumption of a through-thickness crack. For each increment in crack growth, contour integrals and enrichment functions were evaluated in the region around the crack front based on the energy release rate, or J-integrals [42]. Han et al. [43] utilize the J-integral method to compute SIFs, highlighting its effectiveness in integrating energy release rates around the crack tip and delivering highly accurate, mesh-independent results. In Abaqus, the number of contour integrals evaluated is analogous to the number of rings of elements used in the integral calculation. The enrichment radius, which is the radius around the crack tip that identifies the elements used in the calculation of crack singularity for a stationary crack, was set to three times the element characteristic length in the enriched area. This is the default within Abaqus. For each contour integral, the SIFs are provided for each node across the crack front. It is recommended that the number of contour integrals evaluated be chosen based on the convergence of the calculated stress intensity values. A Python script in version 3.8 was developed to incrementally grow the crack and extract the associated SIFs at each increment based on the crack geometry. The geometry and number of cycles between increments in growth were tracked from the images generated during the experimental testing. Within the script, for each image, lists of the crack coordinates were inserted into the model as a through-thickness crack, an analysis was run using the loading conditions of each test, and the SIFs were extracted from the output file. The cycle information, crack lengths, and SIFs were used to generate the data points from which the Paris’ law parameters are estimated. A challenge that arose while implementing XFEM was that if the crack front fell too close to an element node, convergence often did not occur. Because of this limitation, some remeshing was required and incorporated into the Python script if an error message occurred.

### 2.2. Example: Experimental Study and In Situ Miter Gate

The in-service structure under investigation is The Dalles Lock & Dam, designed and operated by the U.S. Army Corps of Engineers, located on the Columbia River in Oregon. Existing physical testing images generated during a previous study by Eick et al. [21] are leveraged by the proposed methodology to provide an a priori estimate of Paris’ law parameters. Then, images acquired of an existing crack on the structure are leveraged to monitor crack growth over time and update the estimated Paris’ law parameters.

#### 2.2.1. Background to Miter Gate Structures

**Figure 4:**
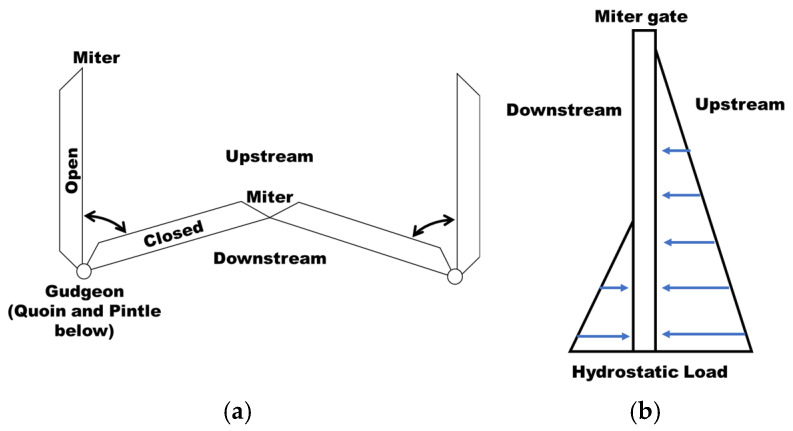
(**a**) Opening and closing of a miter gate and (**b**) application of hydrostatic load.

**Figure 5:**
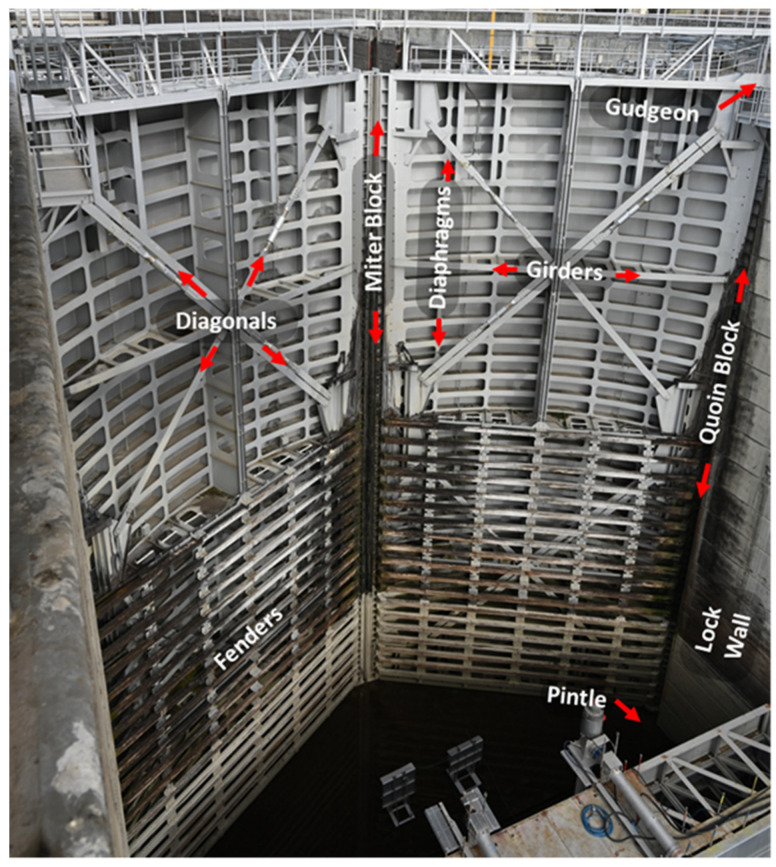
Major components of a miter gate. Miter gate pictured is located at The Dalles Lock & Dam (each leaf is approx. 32.4 m tall, 16.3 m wide).

Miter gates are typically all-welded structures used on either side of lock chambers to allow vessels to pass through a lock site. A loading cycle of a miter gate consists of swinging open and closed to allow vessels to enter or exit, as well as withstanding significant changes in hydrostatic pressure from the raising and lowering of the water level within the lock chamber. These scenarios are illustrated in Figure 4. When a horizontally framed miter gate, as labeled in Figure 5, is hydrostatically loaded, the load path travels through the horizontal girders and into the lock wall. The vertical diaphragms act as stiffeners. The vertical load caused by gravity is primarily carried by the pintle, the system about which the gate rotates.

#### 2.2.2. Paris’ Law Procedures for Laboratory Testing

**Figure 6:**
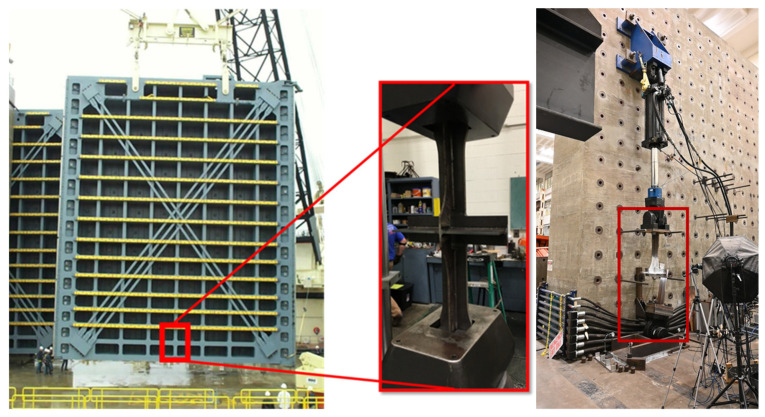
Cruciform specimen representative of the miter gate diaphragm and girder intersection and the full-scale physical test setup using a 220-kip actuator.

Eick et al. [21] performed a study on full-scale cruciform specimens which are representative of a miter gate welded intersection of the vertical diaphragms and the horizontal girders. A total of twelve full-scale specimens were fabricated and tested until failure under constant-amplitude cyclic loading using a 220-kip actuator (Shore Western, Monrovia, CA, United States). The assembled cruciform specimen and test setup and an image demonstrating the specimen’s representative location are shown in Figure 6. Eick et al. [21] detail the full-scale testing methodology and calculation of the associated S-N curve to aid in fatigue life prediction.

**Figure 7:**
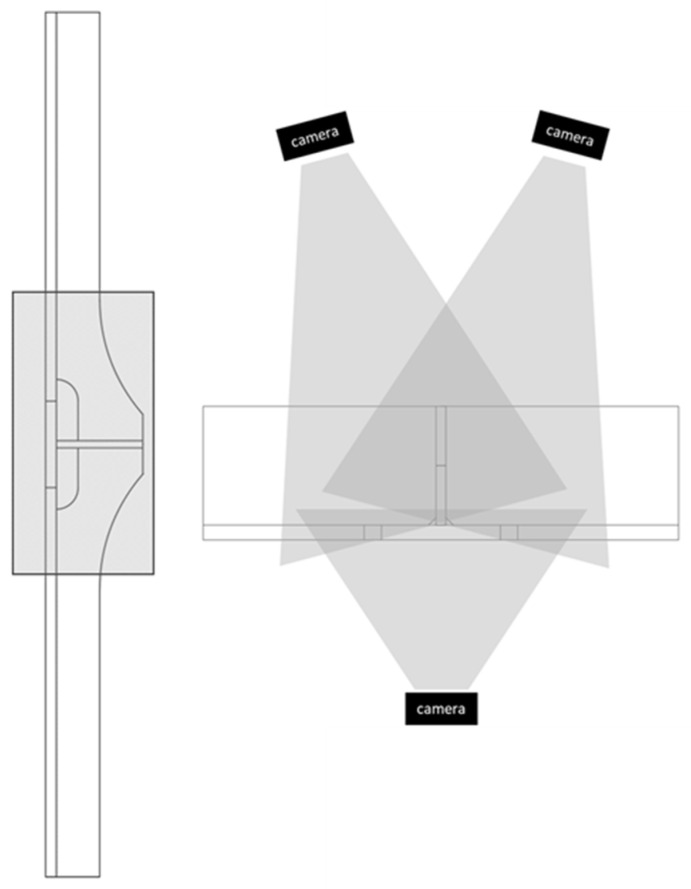
Regions of specimen covered by camera setup. The fourth camera is monitoring the actuator.

Fatigue testing of each specimen lasted for several days, where it would have been infeasible for a person to manually measure crack lengths during testing; therefore, a consistent method for identifying the initiation and growth of a crack was required. The image data generated from these experiments were post-processed to analyze fatigue crack growth using a Paris’ law approach. In order to identify the crack tip location, measure crack lengths, and extract the rate of crack growth from the experimental testing, each specimen was set up to facilitate the use of DIC. To enable DIC, a speckle pattern was applied to each specimen in the region where the crack was expected to form using a commercial DIC speckle pattern application kit. During testing, images were taken of the speckled region of each specimen approximately once every ten cycles. A total of four FLIR Grasshopper3 (GS3-U3-91S6M-C) cameras (FLIR, Wilsonville, OR, United States) were positioned in fixed positions around each specimen: one captured the flat face of the specimen, two were oriented towards the cruciform side on either side of the stem, and one was dedicated to recording the load and cycle information from the readout of the actuator. The orientation of the cameras observing the specimen is illustrated in Figure 7. The cameras were configured to simultaneously trigger at the maximum and minimum load of the load cycle, based on the signal from the load cell on the actuator used during testing.

The images collected during testing were processed to identify the cycle number when the crack initiated and to track the crack length and number of cycles elapsed during subsequent images. DIC data processing was completed using the open-source Digital Image Correlation Engine (DICe) software version 2.0 developed by Sandia National Laboratories. Using parameters selected based on guidance found in Jones and Iadicola [44], a mesh was created on the image surface to estimate displacements and strains. The results were visualized using Paraview 5.10.0 software. The experimental data were originally generated during a research effort to calculate S-N curves, which relate stress amplitude to the number of cycles to failure. However, when repurposing this data to characterize the Paris’ law relationship, it was determined that the resolution of the resulting images was not sufficient to directly calculate the SIFs from the displacement field. Additionally, the complicated geometry prevented the use of analytical SIF solutions. Therefore, a numerical model of the cruciform geometry, with the crack geometry determined from analyzing the experimental results, was used to determine the SIFs at the crack tip for each increment in growth.

**Figure 8:**
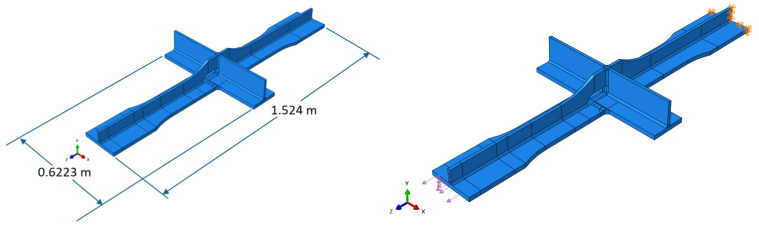
Numerical model of cruciform specimen.

**Figure 9:**
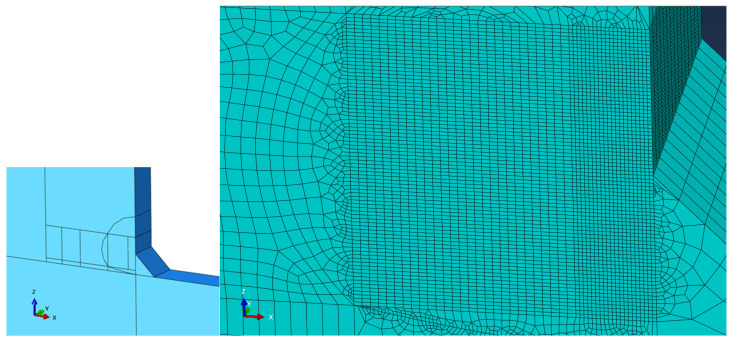
Partitioning and meshing scheme in region where crack initiates.

The numerical model of the fatigue specimen shown in Figure 8 is built in Abaqus using solid geometry. The material is steel with a density of 7.85 g/cm^3^, Young’s modulus of 200 GPa, and Poisson’s ratio of 0.3. The tensile load was applied directly to one end of the cruciform specimen while fixity was applied to the other end. In each of the experimental tests, the crack initiated where the edge of the weld met the base material; therefore, the geometry of the weld was included in the model at this location. The model was partitioned in the region where the crack tended to form to allow for a more focused mesh in the region. This is demonstrated in Figure 9. The minimum mesh seeding size was 0.01, and there are 15 elements (16 nodes) across the thickness of the specimen. This mesh size was required because the crack increments were in the order of 10^−3^ cm (10^−2^ inches). Due to limitations within Abaqus, a crack cannot propagate twice through the same element; therefore, the element size was reduced to avoid errors. The cracks began propagating faster as they approached 2.54 cm (1 inch); therefore, partitioning was used so that the mesh could transition to a larger size as the crack propagated. A total of 140,293 reduced integration hexahedral elements of type C3D8R were generated.

Based on a rigorous convergence study of the SIFs from Abaqus, a total of eight contours were used in the calculation, and SIFs were extracted from the fourth contour. For this study, only the *K_I_* and *K_II_* SIFs were extracted because mode III was negligible in this loading scenario. A Python script was developed to incrementally grow the crack and extract SIFs based on the crack geometry. With the crack growth rates measured from the images, and the associated SIFs estimate from the numerical model, Paris’ law parameters are estimated. The resulting Paris’ law parameters served as the foundation for developing the prior which informed the characterization of crack growth observed on an in situ structure using inspection images.

#### 2.2.3. In Situ Structure Paris’ Law Bayesian Updating

For the in situ miter gate being studied, ten ROV images of a known crack collected over a period of 33 months were utilized. The crack is located on the bottom girder near the pintle region. Each of these images contained varying viewpoints of the crack growing across the girder; therefore, homographic transformations were required to enable comparison of the crack length between images. The coordinates of the discretized crack geometry in each image were noted based on their pixel coordinates. Known distances between image features were used to convert between the pixel and length scales. The crack geometries were then used in a numerical model of an entire miter gate leaf to determine the SIFs at the crack tip for each increment in growth.

**Figure 10:**
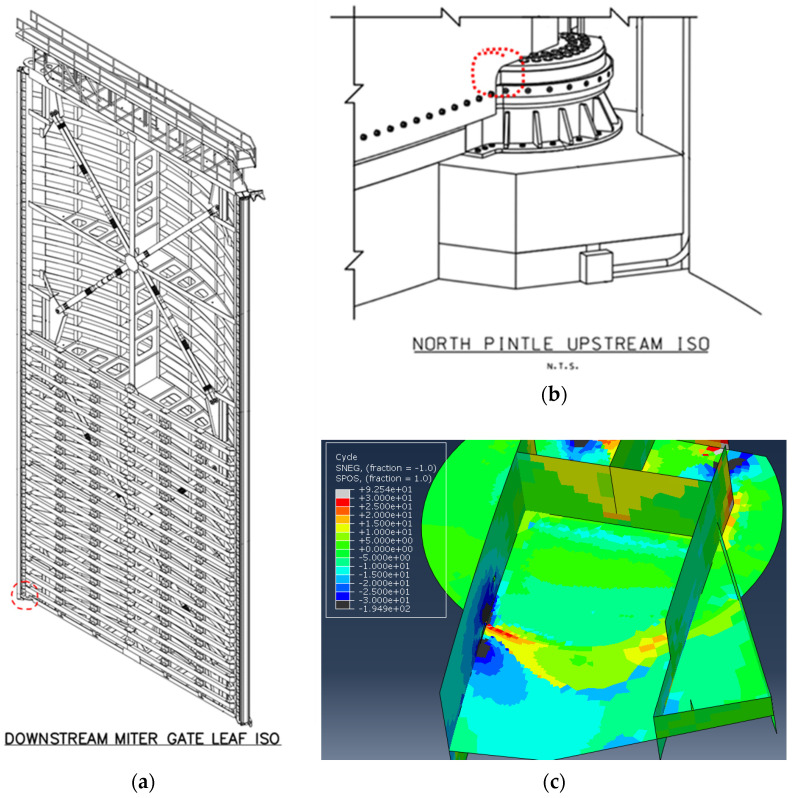
(**a**,**b**) Location of crack initiation near CJP weld in pintle region circled in red; (**c**) difference in signed von Mises stress (ksi) between a gravity loading step and a combined gravity and hydrostatic loading step to indicate a significant change in stress.

The region of the girder where the crack initiated on the miter gate structure is shown in Figure 10. This crack initiated near a complete joint penetration (CJP) weld in the area. Initial inspection of the miter gate numerical model with no crack geometry indicated that the region where the crack initiated experiences significant changes in stress during a loading cycle as shown in Figure 10c. While the geometry and loading conditions of this case differ from those in the experimental testing, both scenarios share the similarities of cracks initiating in mild steel (ASTM A572 Grade 50 in the experiment and ASTM A709 Grade 50 in The Dalles miter gate; these materials are deemed equivalent by the American Railway Engineering and Maintenance-of-Way Association (AREMA) [45]) and occurring in a region in close proximity to a CJP weld. It is expected that the crack growth rate in this location would not exactly follow the results of the experimental testing because of the different materials, environments, state of corrosion, and loading conditions. Therefore, it is necessary to use Bayesian inference to update the initial Paris’ law estimates using the data gathered from conducting remote visual inspections rather than assuming the same behavior and to continuously update the model as more inspections occur.

**Figure 11:**
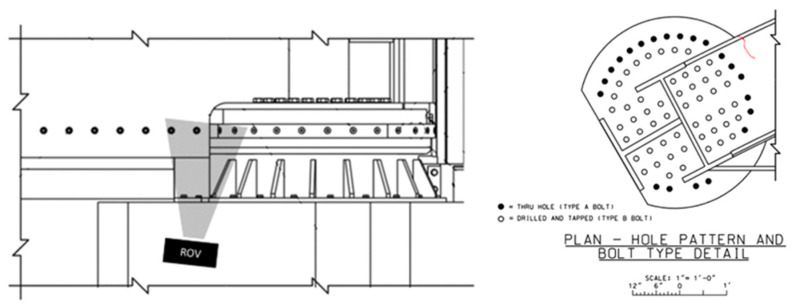
Location and approximate shape of crack on bottom girder.

**Figure 12:**
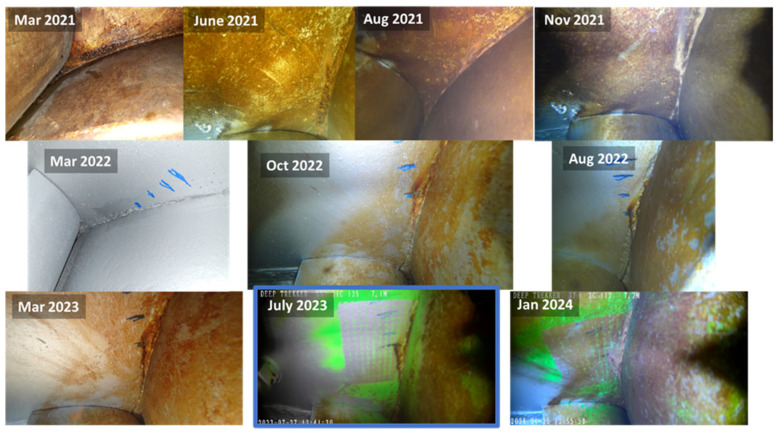
Inspection images: image with blue border (July 2023) contained the most points for comparison.

**Figure 13:**
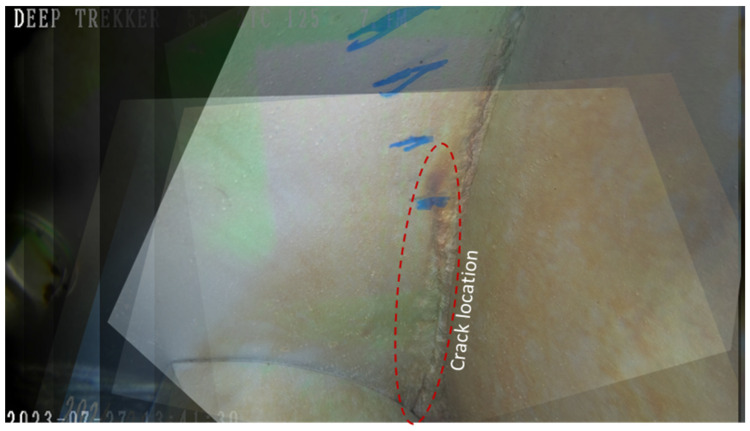
Successfully transformed images overlayed.

Underwater inspections were conducted by engineers using a Deep Trekker DTG3 ROV (Deep Trekker, Kitchener, ON, Canada) with a built-in 8-megapixel camera. The ROV is maneuvered to take images from a view below the gate looking up to the bottom girder as illustrated in Figure 11. In order to compare the crack geometry between subsequent inspections, common points between the inspection images shown in Figure 12 were identified and selected. Defining the perspective of an image requires a minimum of four common points to establish collinearity. Images that did not contain at least four common points were eliminated. This reduced the number of usable images from ten inspections to six. In 2022, blue tick marks were made in the region by inspecting engineers to monitor the direction in which they believed the crack would continue to propagate. These marks proved favorable in aligning subsequent images. To facilitate measurements of the crack length, inspecting engineers painted a green grid in the cracked area in July 2023 during scheduled maintenance when the water was drained from the submerged portion of the lock. With the use of homography techniques, the grid enables direct measurements even for images taken prior to the application of the painted grid. It is noted that although the painted grid was added and used to retroactively obtain crack lengths, any known distance between features can be used to obtain a scale. An image containing an overlay of each inspection image projected into an equivalent image space to show collinearity is illustrated in Figure 13.

The coordinates of the crack geometry were extracted and converted to pixel coordinates. Varying levels of image quality made identifying the location of the crack tip difficult; therefore, multiple points were selected at the crack tip to provide a region of where the tip may be located. These coordinates were then transformed into a flat plane, and finally, the coordinates were converted to physical units using a scaling factor based on the painted grid. For each inspection image, the total visible crack length was calculated from the extracted coordinates to determine the growth between subsequent images. Due to the varying levels of clarity in determining the position of the crack tip, three points were selected and averaged for each image to estimate the crack tip location. This technique was used in cases where shadows or corrosion made identifying the exact pixel difficult. The selected points for averaging were within a few pixels of each other. The crack geometries were then compared to determine which instances resulted in a positive change in total crack length. Using this method, only four instances of crack growth were identified. The four crack geometries were fed into the numerical model of the miter gate, and the SIFs were extracted for each data point. To estimate the number of loading cycles that occurred between each data point, an approximation of five lockages per day was assumed. This was deemed a reasonable estimate by the project engineers.

**Figure 14:**
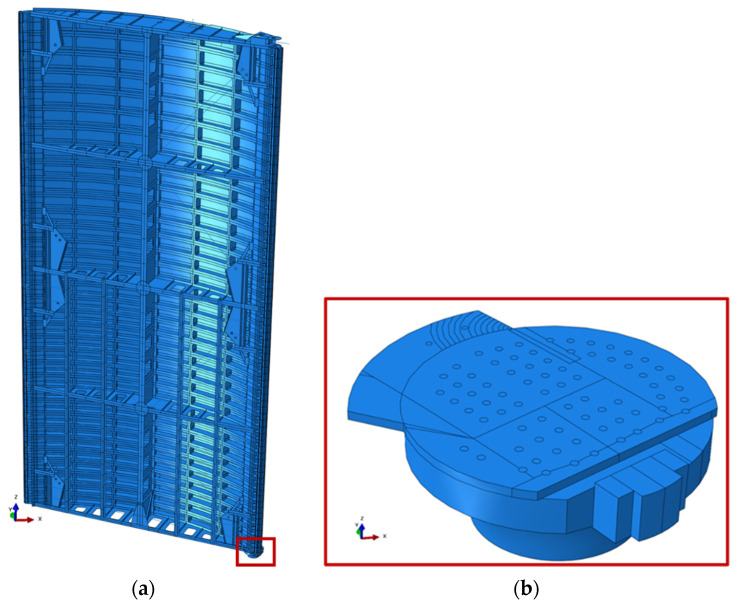
(**a**) Fully assembled miter gate leaf; (**b**) portion of bottom girder tied to pintle region. The solid portion of the girder is connected to the rest of the girder using shell-to-solid coupling.

For the miter gate numerical model shown in Figure 14, a single gate leaf on the downstream end of the lock chamber is modeled using the as-built drawings of The Dalles Lock and Dam provided by engineers. Only a single leaf is represented because symmetry is assumed across the chamber. The material is steel with a density of 7.85 g/cm^3^, Young’s modulus of 200 GPa, and Poisson’s ratio of 0.3. The assembly was made up of a mixture of shell, wire, and solid parts. Constraints between assembled parts were created using tie constraints, multi-point constraints, and shell-to-solid coupling. A global mesh size of 2.0 is applied, with a minimum local seeding of 0.01 used in the crack growth region. A total of 1,209,235 elements were generated for the entire assembly and were a mixture of hexahedral, quadrilateral, triangular, tetrahedral, and line elements. Based on extensive experience with numerical modeling of miter gates, this mesh size is deemed to be sufficiently refined for the accuracy required.

**Figure 15:**
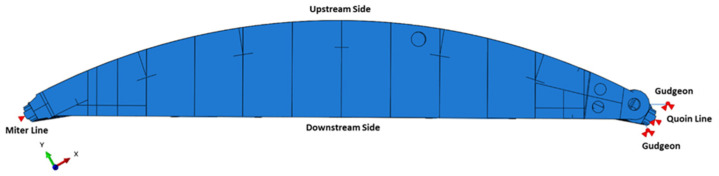
Numerical model boundary conditions.

The load cases applied to the miter gate are a gravity step, where gravity is applied to the entire model, as well as a hydrostatic loading step, to represent the situation where the lock gate is closed and damming the water of a full lock chamber. The hydrostatic load is applied as a differential lateral pressure and is associated with a water level of 32 m (105 feet) on the damming-surface side and a water level of 6.1 m (20 feet) on the other side of the gate. The boundary conditions, constraints, and interactions set within the numerical model follow the general best practices for the pintle, gudgeons, quoin block, and miter block as detailed by Eick [46]. The boundary conditions within the gravity step are the restriction of vertical displacement at the pintle, while at the gudgeons, only rotation about the vertical axis is allowed. During the hydrostatic loading step, contact between the quoin block and lock wall is represented by a lateral boundary condition along the quoin block in the XY plane. Similarly, contact between the miter block and the adjacent gate is represented by a lateral boundary condition along the miter block and perpendicular to its face. These boundary conditions are depicted in Figure 15. The parts of the model are connected in the assembly using tie constraints, MPC beam constraints, or shell-to-solid couplings. The SIFs for all three modes were extracted from the tenth contour integral, when the values converged.

## 3. Results and Discussion

### 3.1. Parameters from Experimental Results

**Figure 16:**
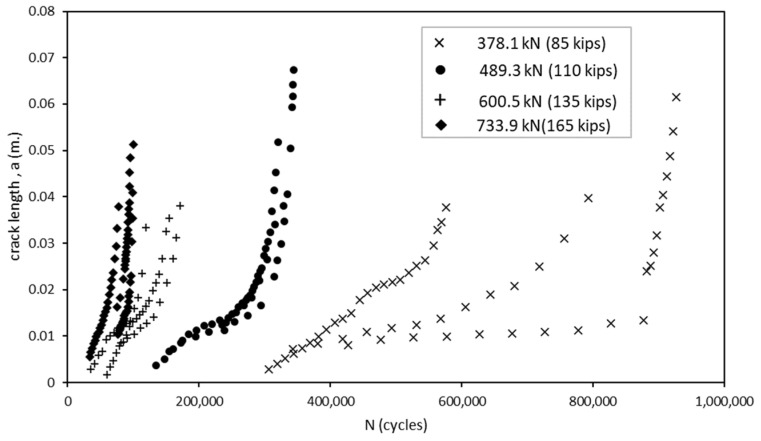
Crack propagation extracted using DIC plotted as crack length vs. number of cycles.

**Figure 17:**
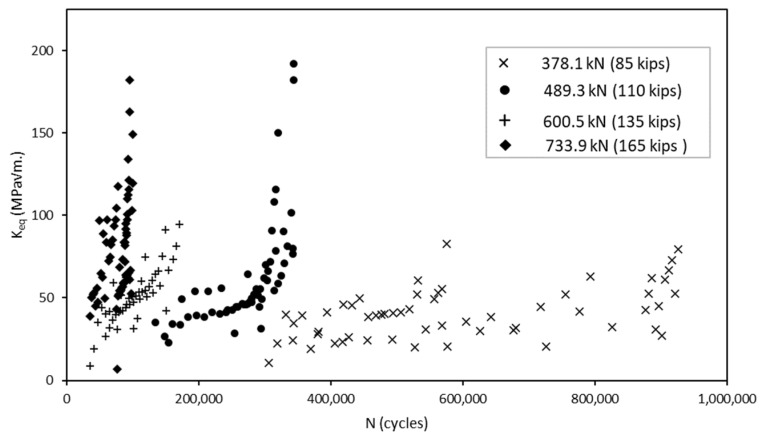
Stress intensity factors extracted from numerical model plotted as K_eq_ versus number of cycles.

**Figure 18:**
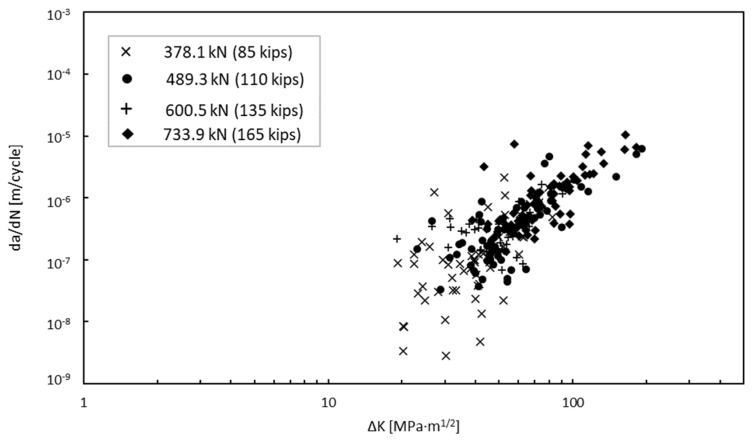
Crack growth rate versus SIF.

To calculate the parameter values, the crack geometry from the experimental testing and load information were inserted into the numerical model in order to extract the associated SIF for each mode of cracking for each increment in growth. Because the load ratio was effectively zero, the stress intensity factors extracted from Abaqus were taken as ∆*K*. Figure 16 presents the results of the experimental testing and crack growth extracted using DIC. For each magnitude of load, the number of cycles until failure stayed within approximately 50,000 cycles of their counterparts with the exception of the specimens loaded at 378.1 kN (85 kips). Figure 17 provides the SIFs derived from the Abaqus FEA model for each increment in the crack growth. By combining the data from these results and converting them to a logarithmic scale, we obtain the relationship shown in Figure 18. Upon first observations, there is an apparent correlation between the crack growth rate and the SIFs.

**Figure 19:**
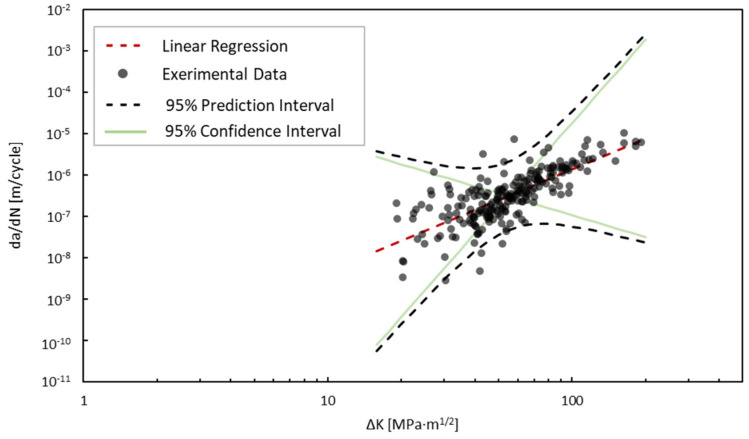
Crack growth rate versus SIF and linear regression of all experimental data.

To linearly fit the log-scale relationship between the crack growth rate and the SIFs defined by Paris’ law, a least squares linear regression approach was applied as described in statistical texts such as those of Ang and Tang [47], Haldar and Mahadevan [48], and Benjamin and Cornell [32]. The Paris’ law parameters found in the experimental study using least squares linear regression are described in Table 1, along with the variance, *σ*^2^, and coefficient of determination, *R*^2^. Each parameter is defined to follow a normal distribution. Based on the calculated coefficient of determination, the linear regression analysis showed a moderate correlation with the log scale of the data. The moderate correlation is indicative of the uncertainty introduced to fatigue crack growth due to welding. Many studies that report crack growth parameters, such as the well-known Virkler study [49], show a high correlation between $\frac{da}{dN}$ and ∆*K*. However, many of these specimens are polished specimens made from a uniform material. The goal of the experimental study was to capture conditions that would be representative of an in situ structure, thus incorporating as much uncertainty as would be expected due to welding, heat-affected zones, and misalignments. Additionally, there is some inherent measurement error in determining the crack geometry. This can especially be seen in the low-load fatigue values, where the increments in crack growth between cycles are orders of magnitude smaller. Using a probabilistic approach enhances these parameter descriptions because of the inherent uncertainty in the crack length measurements. The linear regression results as well as the 95% confidence interval and 95% prediction interval are shown in Figure 19.

**Figure 20:**
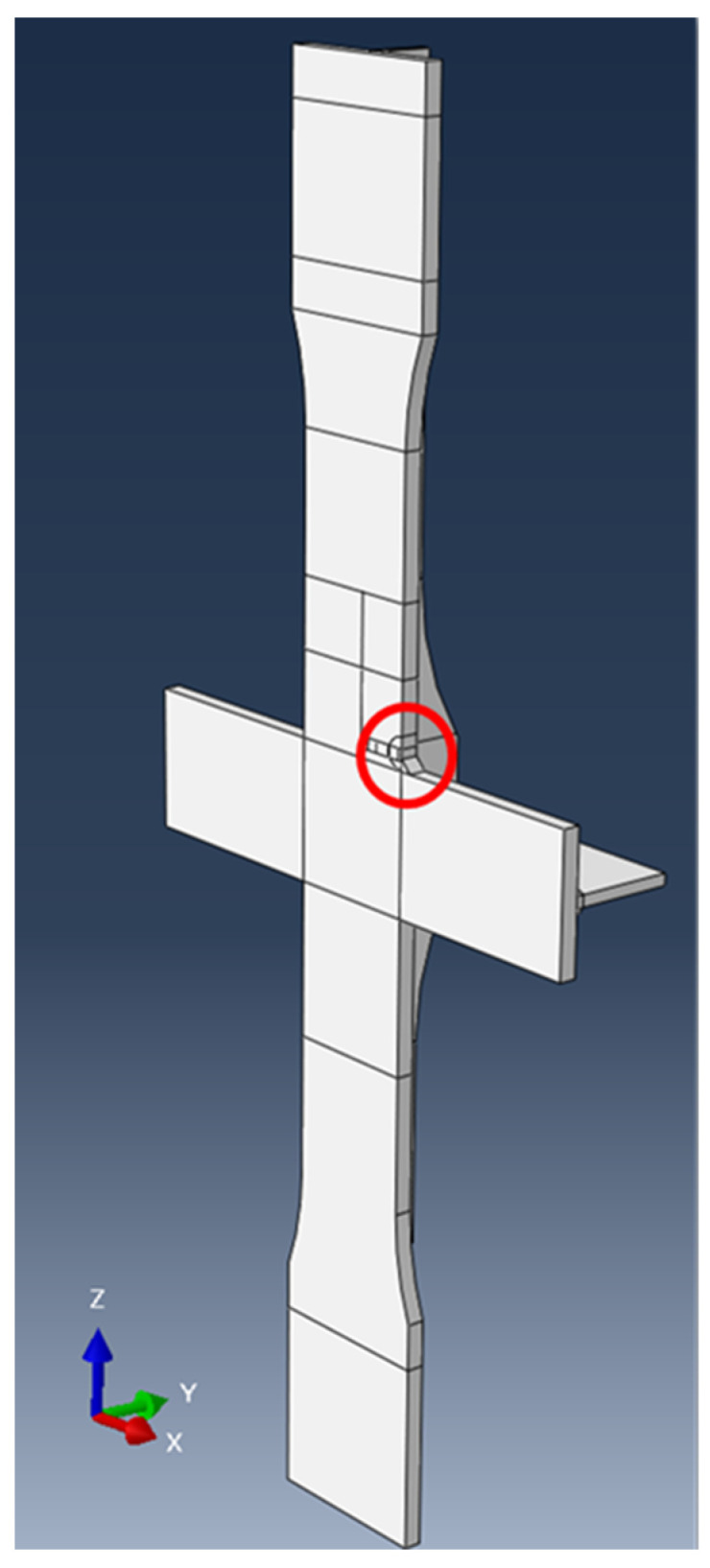
Location of crack growth.

**Figure 21:**
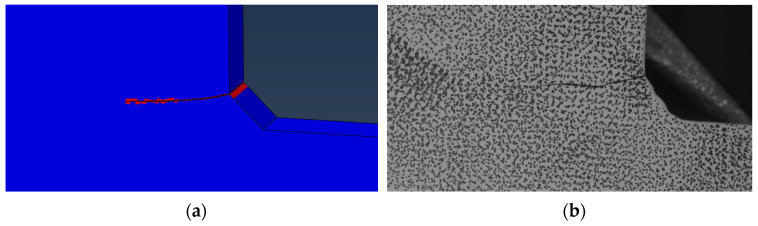
(**a**) Predicted crack growth in ABAQUS using Paris’ law parameters estimated from standard linear regression; (**b**) example specimen from experimental crack growth for a maximum load of 489.3 kN (110 kips).

As an additional measure, to test the predictive accuracy of the obtained parameters, the Paris’ law parameters were used to grow a crack in Abaqus using XFEM on the cruciform specimen. An algorithm was written to incrementally extract the SIF and crack propagation direction based on the maximum tangential stress; however, within the algorithm, the values of the Paris’ law parameters were used as inputs to calculate the number of cycles, *dN*, and grow the crack in increments, *da*, of 0.032 cm (0.025 in) under a max loading of 489.3 kN (110 kips) and load ratio R = 0. The Paris’ law parameters were randomly sampled from the normal distributions obtained from the standard linear regression approach. The crack was inserted at the location circled in Figure 20. The crack growth was set to stop once a crack length of 3.81 cm (1.5 in) was reached because the “failure length” of most of the test specimens occurred between 2.54 and 5.08 cm (1 to 2 in). This was considered the “failure length” to only consider stable crack growth governed by the linear regime of Paris’ law. The field output variable shown in Figure 21a is STATUSXFEM, which shows fully cracked elements. In the experimental data, the three tests completed for a maximum load of 489.3 kN took an average of 340,542.67 cycles to failure, with a standard deviation of 12,640.49 cycles. The crack predicted using the crack growth algorithm and estimated Paris’ law parameters reached the failure length in 303,432.96 cycles. It is noted that within Abaqus, there is a requirement that an initial crack be input within the model in order to utilize the crack analyses. For the predictive model, an initial crack size of 0.032 cm was given; thus, it is expected that the number of cycles to failure will be less than the experimental results due to the additional cycles for initiating the crack. In addition, there is good agreement between the predicted propagation path between the experimental results and the numerical model.

### 3.2. Bayesian Updating of Parameters from Inspection Results

**Figure 22:**
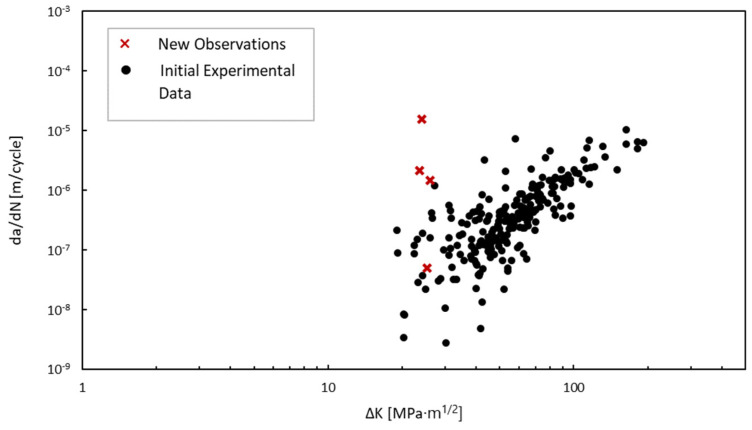
New observations from inspection images with experimental data.

To update the initial experimental Paris’ law parameters, Bayesian inference was used to account for the additional crack progression measurement obtained from the visual inspection images. The data extracted from the images and Abaqus model were processed and added to the experimental dataset as shown in Figure 22. The equivalent change in stress intensity factor was calculated as the difference between a gravity loading step and the application of the hydrostatic load using Equation (3). The linear regression parameters for the original experimental data were treated as the prior in the Bayesian updating scheme. The prior distributions were defined using the values set in Table 1.

**Figure 23:**
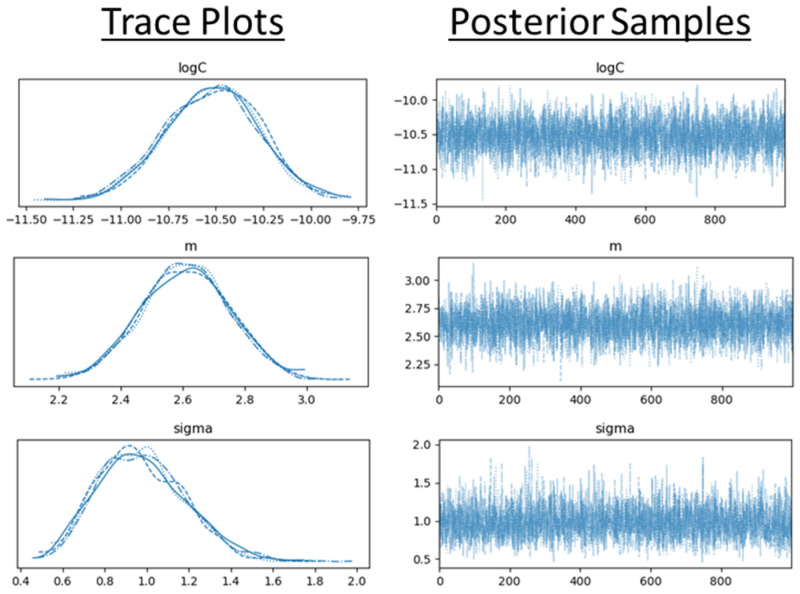
Trace plots and posterior samples from MCMC simulations.

From the analysis of the six usable inspection images, there were only four instances of observable change. These four observations were used to form the marginal and likelihood functions. Although the addition of four observations is a relatively small sample size, it emphasizes the need to collect images of observed cracks in the field. Using the experimental data as the prior within the Bayesian analysis aids in overcoming the limited sample size. Using MCMC simulations, 1000 posterior samples were drawn. These samples were then used to estimate the posterior distributions for each of the model parameters. The trace plots and posterior samples are shown in Figure 23.

**Figure 24:**
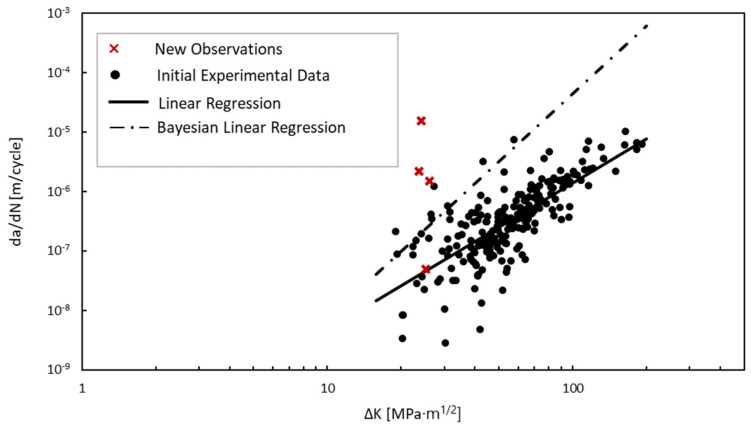
Bayesian linear regression results.

The resulting parameter estimates of the Bayesian updating are shown in Table 2 along with the observation error, σ. The new linear regression fit in Figure 24 shows a clear change in the parameter values, most noticeably in the intercept, log *C*. These updated parameters now incorporate the visual inspections conducted on an in situ miter gate in a region constantly underwater. The increased parameter values indicate a faster crack growth rate on the in situ structure than would be predicted by solely relying on the laboratory experimental data. This can be due to a number of reasons such as an underwater vs. in-air loading environment and the location of the crack on the structure.

**Figure 25:**
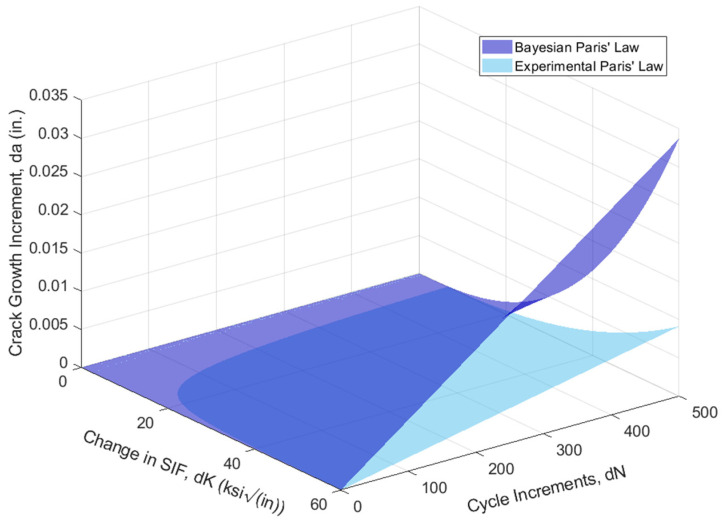
Predicted increment in crack growth (da) for number of cycles (dN) with varying SIF.

Although the relative change in the parameter values seems minor, a small change in each can greatly affect the predicted growth rates. This difference in a predicted crack growth increment, *da*, increases exponentially as the magnitude of the SIF increases for a specified number of cycles between increments in crack growth, *dN*, as demonstrated in Figure 25. For a specified SIF, the change in the predicted crack growth becomes more evident. In comparing the Paris’ law crack growth increment predictions made with the original linear regression parameters with the crack growth predictions made with the updated Bayesian linear regression parameters, for a specified SIF of 54.9 $MPa\sqrt{m}$ (50$\;ksi\sqrt{in}$), the difference in the predicted crack growth reaches almost double the initial estimate. In terms of a time increment, an assumption of five lockages (cycles) per day results in the equivalent of approximately 1828 cycles per year.

Using the numerical model, an average crack growth rate was calculated using the same algorithm to predict the crack growth in the cruciform specimen. The updated parameters resulted in an average crack growth rate of approximately 0.254 mm (0.010 inches) per year. Alternatively, the original parameters resulted in an estimate of approximately 0.031 mm (0.0012 inches) per year, an order of magnitude less than the new predictions. Updating the Paris’ law parameters to more accurately characterize crack growth helps prevent premature failure of structures due to an underestimation of crack growth rates. It is expected that as more data are added to the Bayesian formulation, the predictions of crack growth rates will increase in accuracy.

## 4. Conclusions

The work completed during this study provides a novel methodology for predicting crack growth for an in-service structure, which has important implications for maintenance decisions. While this study utilizes laboratory-tested specimens, any existing Paris’ law data can be utilized as the basis for the proposed framework. Crack growth geometry is extracted from the SIFs from numerical modeling, providing data points necessary to fit Paris’ law. Linear regression of the laboratory data was used to derive the Paris’ law parameters. These parameters serve as a prior within the Bayesian inference formulation for in-service structure crack growth. This methodology was completed by updating the Paris law for an in-service gate with images of the growing crack and numerically calculated SIFs of the crack. Within this methodology, it is assumed that the crack geometries are through-thickness and that images are collected for many increments in crack growth.

This methodology was applied to a case study of a constantly submerged pintle on the miter gate at The Dalles lock. Since the laboratory tests were performed in-air and for a different geometry, updating the Paris’ law parameters was necessary to properly apply them to crack growth in the submerged environment. An ROV collected limited inspection images at the time the study was completed, leading to a sparse dataset; however, the incorporation of Bayesian inference into the methodology allows the parameters to be updated as more inspections occur, and more images are collected. The specific results presented in the paper are expected to only be applicable to miter gates with materials similar to ASTM A572 steel and subjected to similar load scenarios as the gate studied herein. This research finds that the use of images and numerical models provides an effective framework for extracting Paris law information from in-service structures. Further, this research finds that the crack grows an average of 0.254 mm (0.010 inches) per year compared to the original estimate of 0.031 mm (0.0012 inches) per year. The use of the original Paris law parameters would underestimate crack growth, possibly leading to premature failure, unplanned outages, and significant costs. Ultimately, the framework provided herein for incorporating experimental data, numerical modeling, and inspection measurement to assess fatigue crack growth is generalizable to any structure.

The estimates made within this study are based on the cycling of hydrostatic loads. However, in-service miter gates are also subject to additional loads such as opening and closing procedures and thermal stresses from seasonal changes, which are not accounted for in this research. These additional loads have the potential to contribute to additional fatigue cycles. These scenarios will be incorporated into future research by extracting the stress intensity factors from numerical models of these additional loading scenarios to calculate the changes in stress at the crack tip throughout a true loading cycle. Future work will address collecting additional inspection measurements to further update the Paris’ law parameters, while also exploring additional structures on which the proposed methodology can be applied.

## Figures and Tables

**Table 1:** Parameter estimates for Paris’ law using length and SIF units of meters and MPa√meters.

Parameter	Mean	Standard Deviation
*m*	2.477	0.145
log*C*	−10.805	0.253
Other regression parameters		
σ^2^	0.178	
R^2^	0.570	

**Table 2:** Bayesian parameter estimates for Paris’ law using length and SIF units of meters and MPa√meters.

Parameter	Mean	Standard Deviation
*m*	2.605	0.138
log*C*	−10.517	0.243
σ	0.981	0.206

## Data Availability

The original contributions presented in the study are included in the article, further inquiries can be directed to the corresponding author.

## Abbreviations

The following abbreviations are used in this manuscript:

ASTM	American Society for Testing and Materials
CJP	Complete Joint Penetration
DIC	Digital Image Correlation
DICe	Digital Image Correlation Engine
FEA	Finite Element Analysis
FEM	Finite Element Method
GFEM	Generalized Finite Element Method
MCMC	Markov Chain Monte Carlo
MDPI	Multidisciplinary Digital Publishing Institute
NUTS	No-U-Turn Sampler
ROV	Remotely Operated Vehicle
SIF	Stress Intensity Factor
USACE	United States Army Corps of Engineers
XFEM	Extended Finite Element Method
